# Intention to leave Nursing during the COVID-19 pandemic

**DOI:** 10.1590/1518-8345.5815.3549

**Published:** 2022-08-01

**Authors:** Luciane Prado Kantorski, Michele Mandagará de Oliveira, Poliana Farias Alves, Carlos Alberto dos Santos Treichel, Carla Gabriela Wünsch, Luiza Hences dos Santos, Guilherme Emanuel Weiss Pinheiro

**Affiliations:** 1 Universidade Federal de Pelotas, Faculdade de Enfermagem, Pelotas, RS, Brasil.; 2 Bolsista do Conselho Nacional de Desenvolvimento Científico e Tecnológico (CNPq), Brasil.; 3 Universidade Estadual de Campinas, Faculdade de Ciências Médicas, Campinas, SP, Brasil.; 4 Universidade Federal de Mato Grosso, Faculdade de Enfermagem, Cuiabá, MT, Brasil.; 5 Bolsista da Coordenação de Aperfeiçoamento de Pessoal de Nível Superior (CAPES), Brasil.; 6 Universidade Federal de Santa Maria, Colégio Politécnico, Santa Maria, RS, Brasil.

**Keywords:** Nursing, Nursing Team, Pandemics, COVID-19, Health Personnel, Burnout Psychological, Enfermagem, Equipe de Enfermagem, Pandemias, COVID-19, Pessoal de Saúde, Esgotamento Psicológico, Enfermería, Grupo de Enfermería, Pandemias, COVID-19, Personal de Salud, Agotamiento Psicológico

## Abstract

**Objective:**

to investigate the percentage of professionals with an intention to leave Nursing during the COVID-10 pandemic, as well as the factors associated with this outcome.

**Method:**

a cross-sectional study conducted by applying questionnaires to 890 Nursing professionals from the municipality of Pelotas (RS). The outcome was identified by means of self-reports obtained from the question itself. Relative Risks, as well as their Confidence Intervals (95%), were calculated for the independent variables by means of unadjusted and adjusted Poisson regression.

**Results:**

the percentage of professionals who stated their intention to leave Nursing was 24.6% (n=219). There was a positive association between the outcome and higher schooling levels, negative evaluation of institutional support, moderate or intense overload, and skin lesions. A negative association was also observed between the outcome and individuals aged 51 years old or more.

**Conclusion:**

except for skin lesions, aspects such as lack of support and overload, although they may have been intensified during the pandemic, do not represent a new fact in the health services. In this sense, the associations found in the study reflect the need for cross-sectional actions to promote retention of professionals.

## Resumo

**Objetivo:**

investigar a proporção de profissionais com intenção em deixar a Enfermagem durante a pandemia de COVID-19, bem como os fatores associados a esse desfecho.

**Método:**

estudo transversal realizado por meio da aplicação de questionários a 890 profissionais de Enfermagem do município de Pelotas (RS). O desfecho foi identificado por meio do autorrelato obtido a partir de questão própria. Riscos Relativos, bem como seus Intervalos de Confiança (95%), foram calculados para as variáveis independentes por meio de regressão de Poisson bruta e ajustada.

**Resultados:**

a proporção de profissionais que manifestaram intenção em deixar a Enfermagem foi de 24,6% (n=219). Houve associação positiva entre o desfecho e maior escolaridade, avaliação negativa do suporte institucional, sobrecarga moderada ou pesada e lesões de pele. Foi observada ainda associação negativa entre o desfecho e idade igual ou superior a 51 anos.

**Conclusão:**

exceto pelas lesões de pele, aspectos como a falta de suporte e a sobrecarga, embora possam ter sido exacerbadas na pandemia, não caracterizam fato novo nos serviços de saúde. Nesse sentido, as associações encontradas pelo estudo refletem a necessidade de ações transversais para promover a retenção de profissionais.

## Resumen

**Objetivo:**

investigar el porcentaje de profesionales con intención de abandonar la profesión de Enfermería durante la pandemia del COVID-19, así como los factores asociados con este desenlace.

**Método:**

estudio transversal realizado por medio de cuestionarios aplicados a 890 profesionales de Enfermería del municipio de Pelotas (RS). El desenlace se identificó por medio de autoinformes obtenidos a partir de la pregunta en sí. Por medio de regresión de Poisson ajustada y no ajustada, se calculó el Riesgo Relativo, al igual que sus Intervalos de Confianza (95%), para las variables independientes.

**Resultados:**

el porcentaje de profesionales que manifestaron su intención de abandonar la profesión de Enfermería fue del 24,6% (n=219). Se registró una asociación positiva entre el desenlace y nivel de estudios más elevado, evaluación negativa del apoyo institucional, sobrecarga moderada o intensa y lesiones en la piel. También se observó una asociación negativa entre el desenlace y personas de al menos 51 años de edad.

**Conclusión:**

salvo por las lesiones en la piel, aspectos como la falta de apoyo y la sobrecarga, aunque pudieran haberse visto intensificados durante la pandemia, no representan un hecho novedoso en los servicios de salud. En este sentido, las asociaciones que se encontraron en el estudio reflejan la necesidad de implementar acciones transversales para promover retención de profesionales.

**Highlights**

(1) Positive association with Higher Education schooling level and skin lesions during the pandemic.

(2) Positive association with a deficient evaluation of the support received in the health service.

(3) Positive association with moderate or intense work overload.

(4) Need for cross-sectional actions to stay in the profession.

(5) The findings contribute to guiding the public policies.

## Introduction

Nursing represents more than half of the workforce in the Brazilian health system and, in June 2021, assistants, technicians and nurses accounted for 2,521,155 professionals^1^. Factors such as lack of support from the employer, lack of autonomy, low remuneration, increased workload and demotivation, in addition to physical and mental illness, have marked the work context of the Nursing team^2^ ^-^ ^6^.

On January 30^th^, 2020, the World Health Organization announced the outbreak of the Novel Coronavirus (Severe Acute Respiratory Syndrome Coronavirus 2 - SARS-CoV-2) as a Public Health Emergency of International Concern, declaring it a pandemic on March 11^th^, 2020. In Brazil, the first case was identified on February 26^th^, 2020, with rapid dissemination of the virus and an increasing search for care and overload in the health services. The initial scenario of lack of knowledge about the virus, the scarcity of adequate Personal Protective Equipment (PPE) items and testing and nonexistence of a vaccine and treatment protocols, added to the need to expose themselves to contagion and the greater confrontation to risks of contamination in the work of the Nursing team, accentuated the already existing difficulties^7^ ^-^ ^8^. Until early September 2021, 4,534,755 lives had been lost worldwide^9^; among them, nearly 3,000 were Nursing professionals^7^, with 864 in Brazil alone^10^. 

The scarcity of Nursing professionals noticed in many countries and dissatisfaction with the working conditions were aggravated during the COVID-19 pandemic. The current context, marked by deaths, illnesses and an increase in workload, has influenced these workers’ intention to leave the profession and, in turn, exerted an impact on the service provided to the population^8^ ^,^ ^11^.

A cross-sectional, observational and multicenter study conducted before the COVID-19 pandemic with 23,159 professionals from the Nursing team of surgical and medical units of 385 hospitals in ten European countries (Belgium, Finland, Germany, Ireland, Netherlands, Norway, Poland, Spain, Switzerland and the United Kingdom), found that, in general, 9% intended to leave the profession (varying from 5% to 17% across all ten countries). A more negative perception about their participation in hospital affairs, older age, female gender, full-time work and Burnout syndrome were associated with the intention to leave Nursing^12^. More recently, in the pandemic context, a cross-sectional study conducted with 385 professionals from the Nursing team in community services in the Philippines showed that those who expressed fears of working in the face of the challenges inherent to the COVID-19 pandemic in the multivariate analysis were more likely to suffer from stress at work and to have an intention to leave the profession^13^. 

It is noted that there are gaps in the literature about the factors that contribute for Nursing workers reporting their intention to leave the profession^3^ ^-^ ^4^ ^,^ ^13^. Studying these factors can contribute to planning the workforce, improving the working conditions and facing the difficulties, in addition to developing management strategies and practices so that these workers do not leave the profession.

It is believed that the intention to leave the Nursing profession is associated with factors related to the working conditions and that it has been potentiated in the COVID-19 pandemic context. Consequently, the objective of this study was to investigate the percentage of professionals with an intention to leave Nursing during the COVID-19 pandemic, as well as the factors associated with this outcome.

## Method

### Study type and design

This is a cross-sectional study conducted during June and July 2020 in the municipality of Pelotas (RS). The study population comprised Nursing workers (nurses, nursing technicians and nursing assistants) allocated in the municipal services aimed at coping with the COVID-19 pandemic, namely: 51 basic health units; two outpatient services; two hospital services; an emergency service; a mobile emergency service and a teleconsultation service, in addition to the municipal epidemiological surveillance service and the vacancy regulation center. According to a previous survey, the total number of Nursing professionals linked to these services was 1,297. 

### Selection of the participants

The following inclusion criteria were adopted for this study: being over 18 years of age; being a Nursing professional duly registered at the Regional Nursing Council (*Conselho Regional de Enfermagem*, COREN) and being active in the services to fight against the COVID-19 pandemic in the municipality under study. The exclusion criteria were the following: being on vacation or distanced from work activities during the data collection period and not having a valid contact means (email address; messaging app; cell phone or landline) registered with the services under study.

### Data collection procedures

After applying the inclusion and exclusion criteria, a total of 1,186 eligible professionals were identified for the study. Among these, 944 successful contacts were made, with 242 cases in which, after at least ten attempts on different days and times, it was not possible to contact the professionals. Contacts with the participants were in charge of 18 undergraduate or graduate students attending the Nursing or Psychology courses, previously trained to follow the study protocol (approach means and method; clarification of the study procedures and objectives; ethical issues). They were supervised by professors or researchers with a PhD and previous experience in the conduction of research studies.

Among the successful contacts, and after clarification regarding the study objectives and the right for non-participation and anonymity, 54 professionals refused to participate in the study. Consequently, 890 professionals were included in the study, corresponding to a response rate of 75% of the eligible population.

Those who agreed to participate in the study received, through their email address, Short Message Service (SMS) or personal messaging app, a link that directed them to a self-applied online questionnaire, hosted on the Google Forms platform, which could only be accessed after reading and agreeing with the Free and Informed Consent Form (FICF).

### Quality control

The questionnaires received were reviewed daily by the supervisors in order to identify those that were only partially answered or other issues that could invalidate them, such as if they had been answered by professionals who were not included in the lists provided by the services. 

Each participant was instructed to answer the questionnaire only once, even if they worked in more than one service. In these cases, they were asked to answer the questionnaire thinking about the job they considered as their main employment contract.

### The outcome and its measurement

The intention to leave Nursing was assessed through a question elaborated and validated by the study researchers in a pilot test with ten Nursing professionals, who did not comprise the study sample. The question asked the following to the professional: “Did you think about changing professions at some moment during the COVID-19 pandemic?”. Presence of the intention to leave Nursing was considered when the professionals gave a positive answer to this question.

### Independent variables

The questionnaire applied to the participants was self-prepared and took into account a previously conducted literature review. From this review, questions were elaborated about the sociodemographic profile, training, work process (in general and related to the pandemic), support, changes in the routine and impacts of the pandemic.

The following were included as independent variables in this study: gender (male and female); skin color (white, brown, black); age (up to 30, from 31 to 40, from 41 to 50, 51 or more); schooling (High School, graduate level, undergraduate level); *per capita* income (up to one minimum wage, up to two minimum wages, up to three minimum wages, more than three minimum wages); belonging to a risk group (characterized by the presence of comorbidities such as hypertension, diabetes, and heart or respiratory chronic diseases, in addition to a history of transplantation or use of immunosuppressants - does not belong or belongs); Nursing category (Nurse, Nursing Technician, Nursing Assistant); type of service (Primary Health Care, outpatient, emergency, hospital, administrative); assessment of the working conditions (good, fair, deficient); evaluation of the institutional support received (good, fair, deficient); overload (light, moderate, intense); comparison between pre- and post-pandemic overload (unchanged/reduced, increased); degree of involvement with actions to fight against the pandemic (none, indirect work - for example: administrative, contact with suspected cases, contact with confirmed cases); training to cope with the pandemic (no, yes); lack of PPE (no, yes); presence of skin lesions (no, yes); suspected contagion (no, yes); COVID-19 cases in the family or close friends (no, yes); reduced interaction with family members (no, yes).

### Statistical analyses

The statistical analyses were performed in the *Stata*16 software (*Stata Corporation*, College Station, Texas, USA). The prevalence values of the outcome were calculated between the sample and within each stratum of the variables under study.

The associations between the outcome and the independent variables were tested using unadjusted and adjusted Poisson regression models with robust variance estimators. In the adjusted analysis, the *stepwise forward* technique was employed for selection of the potential confounding factors, adopting p-value ≤0.20 as selection criterion^14^. Statistical significance was defined as p < 0.05.

After selection, the “skin color”, “Nursing category”, “evaluation of the institutional support received”, “overload”, “skin lesions” and “reduced interaction with family members” variables were defined as potential confounders. In this sense, they were used for adjustment between each other and for each of the other variables included in the study. 

It is emphasized that the regression analysis was preceded by a collinearity test and that the results were satisfactory to perform the analyses (Variance Inflation Factor -VIF < 1.36).

### Ethical procedures

The study was submitted to and approved by an accredited Committee of Ethics in Research involving human beings, under Technical Opinion No. 4,047,860 of May 26^th^, 2020, following the Brazilian rules regarding regulation and guidelines for research involving human beings (CNS Resolution No. 466/2012), in addition to the Declaration of Helsinki. The ethical principles of this study were observed by guaranteeing the right not to participate in the research from the first contact and by adopting the FICF in which, by accepting, the participant agreed with data disclosure for scientific purposes, preserving anonymity.

The Strengthening the Reporting of Observational Studies in Epidemiology (STROBE) guidelines were complied with in this study.

## Results

In total, 890 Nursing professionals answered the online questionnaire. Of these, 319 (35.8%) were nurses, 501 (56.3%) were nursing technicians and 70 (7.86%) were nursing assistants. Most of the participants were female (84.8%; n=755) and the mean age was 40.4 years old (SD=8.58). Regarding the workplace, the majority worked in hospital services (64.8%; n=577) or in Primary Care (10.3%; n=92). The participants’ full characterization can be seen in Table 1. The variable with missing data was *per capita* income (n=80). 

**Table 1:** Sociodemographic data of the study participants (n=890), type of service where they work, and Nursing category to which they belong. Pelotas, RS, Brazil, 2020

Variables	n	%
*Sex*		
Female	755	84.8
Male	135	15.2
*Skin color*		
White	665	74.7
Brown	122	13.7
Black	103	11.6
*Age in years old*		
Up to 30	117	13.2
31-40	365	41.0
41-50	292	32.8
51+	116	13.0
*Schooling*		
High School	330	37.1
Undergraduate level	348	39.1
Graduate level	212	23.8
Per capita income in minimum wages*		
Up to 1	205	25.3
Up to 2	305	37.7
Up to 3	132	16.3
More than 3	168	20.7
*Type of service*		
Primary Health Care	118	13.3
Outpatient	92	10.3
Emergency	84	9.5
Hospital	577	64.8
Administrative	19	2.1
*Nursing category*		
Nurse	319	35.8
Nursing Technician	501	56.3
Nursing Assistant	70	7.9

*Minimum wage in force = R$ 1,045.00 a month (US$ 198)

Of the 890 Nursing professionals, 24.6% (n=219) stated their intention to leave the profession. The prevalence of the outcome stratified by the participants’ Nursing category was 29.1% for Nurses, 22.9% for Nursing Technicians and 15.7% for Nursing Assistants. The proportion of this outcome in relation to the sociodemographic variables and the unadjusted and adjusted Relative Risk values in relation to the other variables included in the study are presented in Table 2.

**Table 2:** Prevalence values of the outcome among the study participants (n=890) according to the Adjusted and Unadjusted Relative Risk and Confidence Interval (95% CI) variables estimated by means of Poisson regression. Pelotas, RS, Brazil, 2020

Variables	n	%	Unadjusted RR (95% CI)	p-value	Adjusted RR* (95% CI)	p-value^†^
*Sex*						
Female	755	24.9	1		1	
Male	135	22.9	0.92 (0.66-1.28)	0.634	0.96 (0.71-1.31)	0.837
*Skin color*						
White	665	25.9	1		1	
Brown	122	23.8	0.91 (0.65-1.29)	0.63	0.83 (0.60-1.16)	0.293
Black	103	14.5	0.67 (0.43-1.04)	0.08	0.73 (0.48-1.10)	0.137
*Age in years old*						
Up to 30	117	30.8	1		1	
31-40	365	27.1	0.88 (0.64-1.21)	0.44	0.90 (0.67-1.20)	0.495
41-50	292	23.3	0.75 (0.53-1.06)	0.111	0.90 (0.65-1.26)	0.570
51+	116	13.8	0.44 (0.26-0.76)	0.003	0.58 (0.35-0.98)	0.042*
*Schooling*						
High School	330	20.0	1		1	
Undergraduate level	348	25.3	1.26 (0.95-1.67)	0.102	0.98 (0.66-1.45)	0.933
Graduate level	212	30.6	1.53 (1.14-2.06)	0.005	1.40 (1.04-1.89)	0.026*
*Per capita income in minimum wages*						
Up to 1	205	22.4	1		1	
Up to 2	305	24.9	1.11(0.80-1.53)	0.522	1.01 (0.74-1.38)	0.929
Up to 3	132	27.9	0.97 (0.64-1.47)	0.92	0.84 (0.55-1.27)	0.423
More than 3	168	23.8	1.06 (0.73-1.53)	0.755	0.77(0.52-1.16)	0.220
*Risk group*						
Does not belong	606	24.9	1		1	
Belongs	284	23.9	0.96 (0.74-1.23)	0.754	0.85 (0.66-1.08)	0.193
*Nursing category*						
Nurse	319	29.1	1		1	
Nursing Technician	501	22.9	0.78 (0.62-0.99)	0.046	0.81 (0.64-1.01)	0.073
Nursing Assistant	70	15.7	0.53 (0.30-0.95)	0.033	0.70 (0.40-1.21)	0.206
*Type of service*						
Primary Health Care	118	25.4	1		1	
Outpatient	92	20.6	0.81 (0.48-1.34)	0.421	0.92 (0.56-1.49)	0.741
Emergency	84	20.2	0.79 (0.47-1.34)	0.395	0.79 (0.47-1.33)	0.391
Hospital	577	26.3	1.03 (0.73-1.45)	0.827	1.03 (0.75-1.42)	0.835
Administrative	19	5.3	0.20 (0.29-1.43)	0.11	0.25 (0.03-1.66)	0.152
*Assessment of the working conditions*						
Good	325	16.3	1		1	
Fair	409	24.9	1.52 (1.13-2.06)	0.005	1.13 (0.78-1.64)	0.502
Deficient	156	41.0	2.51 (1.84-3.43)	0.000	1.26 (0.80-1.98)	0.305
*Evaluation of institutional support*						
Good	263	15.2	1		1	
Fair	363	22.3	1.46 (1.03-2.06)	0.029	1.22 (0.87-1.72)	0.230
Deficient	264	37.1	2.44 (1.76-3.38)	0.000	1.64 (1.17-2.30)	0.004*
*Sensation of overload*						
Slight	343	11.4	1		1	
Moderate	262	23.3	2.04 (1.41-2.95)	0.000	1.71 (1.17-2.49)	0.006*
Intense	285	41.7	3.67 (2.65-5.08)	0.000	2.76 (1.95-3.9)	0.000*
*Comparison of the overload (after COVID-19)*						
Unchanged/Reduced	337	14.2	1		1	
Increased	553	30.9	2.17 (1.62-2.90)	0.000	1.18 (0.85-1.65)	0.306
*Involvement with COVID-19 cases*						
None	288	23.2	1		1	
Indirect work (e.g.: administrative)	65	20.0	0.85 (0.50-1.46)	0.576	0.79 (0.47-1.34)	0.399
Contact with suspected cases	310	23.2	0.99 (0.74-1.33)	0.991	0.86 (0.65-1.13)	0.285
Contact with confirmed cases	277	29.5	1.26 (0.94-1.69)	0.109	0.95 (0.70-1.27)	0.744
*Training to fight against COVID-19*						
No	319	22.9	1		1	
Yes	571	25.6	1.11 (0.87-1.42)	0.376	1.08 (0.85-1.36)	0.496
*Lack of Personal Protective Equipment*						
No	508	21.6	1		1	
Yes	382	28.5	1.31 (1.04-1.65)	0.018	1.13 (0.90-1.42)	0.262
*Skin lesions*						
No	393	15.0	1		1	
Yes	497	32.2	2.14 (1.64-2.80)	0.000	1.60 (1.22-2.11)	0.001*
*Suspected contagion*						
No	574	21.1	1		1	
Yes	316	31.0	1.47 (1.17-1.84)	0.001	1.14 (0.91-1.42)	0.239
*COVID-19 cases in the family or close friends*						
No	616	24.5	1		1	
Yes	274	24.8	1.01 (0.78-1.29)	0.922	0.85 (0.67-1.08)	0.197
*Reduced contact with family members*						
No	301	20.3	1		1	
Yes	589	26.8	1.32 (1.01-1.71)	0.035	1.22 (0.95-1.56)	0.115
*TOTAL*	890	24.61				

*Adjusted for skin color, Nursing category, evaluation of the institutional support received, overload, skin lesions and reduced interaction with family members; ^†^Significance level < 0.05

Regarding the sociodemographic data, evidence of a negative association was found between the intention to leave Nursing and being at least 51 years old (RR: 0.58; 95% CI: 0.35-0.98). On the other hand, evidence of a positive association between the outcome and graduate schooling level was observed, with High School education as a comparison stratum (RR: 1.40; 95% CI: 1.04-1.89).

Evidence of an association between the outcome and the worst evaluation of the institutional support received at work was also found. Those who evaluated the support received in the worst way presented an RR of 1.64 (95% CI: 1.17-2.30) for the outcome in relation to those who assessed it as positive.

The reports of moderate (RR: 1.71; 95% CI: 1.17-2.49) or intense (RR: 2.76; 95% CI: 1.95-3.9) overload also presented a positive association with the outcome, with a higher relative risk of presenting the outcome the more intense the overload. 

An association was also evidenced between the intention to leave Nursing and the emergence of skin lesions as a consequence of using PPE during the pandemic period (RR: 1.60; 95% CI: 1.22-2.11).

## Discussion

This study evidenced a high percentage of Nursing professionals with an intention to leave the profession. This outcome was associated with schooling level, lack of institutional support and moderate and intense workload, with an emphasis on those who presented skin lesions due to PPE use. 

The intention to leave the profession during the COVID-19 pandemic was also found in a study conducted with 210 Nursing professionals in hospitals from Zagazig, Egypt, where more than 95% had the intention to leave their current job in a hospital for COVID-19 screening, while around 25% intended to leave the profession altogether^15^.

In the initial stage of the pandemic in South Korea, a research study conducted with 377 professionals from the Nursing team evidenced that more than 10% of the participants reported their intention to leave the profession^16^. Likewise, the results of a survey involving 1,705 front-line Nursing professionals in Quebec showed that, during the COVID-19 pandemic, 29.5% of the Nursing team members had the intention to leave their current work environment and 22.3%, the profession^17^, as well as Nursing professionals from Australian emergency services, where 20.3% of the 398 professionals studied had the intention to leave the profession^18^.

A research study conducted with 512 Nursing professionals in Qatar found an increase from 13.24% to 15.54% in the intention to leave the job during the COVID-19 pandemic. Although there are differences between the intention to leave the job and look for another one with less exposure and the intention to leave the profession, it is important to realize that this intention can be a fact that precedes leaving the profession^19^. 

In addition to that, in a report from the United Kingdom, it was evidenced that 20% of the national associations of Nursing professionals reported an increase in the number of professionals leaving the profession as a result of the pandemic^12^. It was observed that the prevalence found in this study remained close to or above the values detected in research studies conducted before and during the pandemic.

In this research, it was noticed that Nursing professionals aged at least 51 years old are less likely to leave the profession. These results are corroborated by a study in South Korea, where it was verified that Nursing professionals aged 41 years old or more are engaged at work and with a stronger intention to stay in Nursing.

The results also pointed out that the outcome was associated with the stratum referring to people with higher schooling levels. However, in a study conducted with Nursing professionals in Qatar, the researchers concluded that education qualifications did not interfere in this intention^19^.

In opposition to what was observed in other studies, the results of this research indicate that lack of PPE was not associated with the intention to leave the profession. In a research study carried out with Nursing professionals in the United States, it was found that stress and anxiety were present in 89% of the participants who heard news about the scarcity of PPE. In addition to that, lack of PPE was considered as with a positive relationship with the intention to leave the profession^20^. In Israel, one third of the Nursing professionals reported fearing going to work due to potential contamination and to feeling inadequately protected in the work environment, reasserting the importance of PPE. However, when asked if they regretted choosing the Nursing profession after the pandemic, the interviewees strongly disagreed with this assertion^21^.

Also in contrast to other studies, no statistically significant differences were found between the type of service in which the professionals worked and the intention to leave the profession. In the Philippines, during the COVID-19 pandemic, community service nurses, even with moderate levels of job satisfaction, indicated their intention to leave their jobs and change professions^13^.

The results of the present investigation pointed out that the Nursing professionals who presented intense and moderate workloads had deficient support in the workplace and that those who presented skin lesions were more likely to state their intention to leave Nursing.

It is noteworthy that work overload has been widely discussed in the literature even before the pandemic context. The high level of demands, especially work overload and Burnout, contributed to the intention to leave Nursing^12^ ^,^ ^22^. 

Before the pandemic context, in Poland it was identified that the most important factor for the overall mental health status of the category was work overload. In the study in question, 66.5% (n=371) of the Nursing professionals who worked in hospitals assessed work overload as a negative aspect of the service^23^.

A survey conducted with Nursing professionals in the United States (n=50,273) and based on data collected in 2018 evidenced that 9.5% of the interviewees reported having effectively resigned from their most recent positions and, of these, 31.5% reported exhaustion as a contributing reason for the decision to leave the job. In relation to the Nursing professionals who thought about making this decision, 43.4% identified Burnout syndrome as a reason. Additional factors in these decisions were a stressful work environment, inadequate staffing, absence of good management or leadership and the need for better remuneration and/or benefits^24^.

A research study that compared two groups of Nursing professionals from hospitals in Egypt pointed out that the main contributing factor to stress was the work overload evidenced by 98.6% of the workers in a hospital for COVID-19 screening, in relation to 51.4% of the workers from a general hospital^15^. This overloaded work environment contributes to dissatisfaction at work and can lead to leaving the profession.

In relation to deficient institutional support, corroborating the findings of this study, a survey conducted with Nursing professionals in the United Kingdom related the intention to leave Nursing with lack of support in the work environment. The result pointed out that those Nursing workers who lacked support at work had 4.8 times more chances of feeling dissatisfied^25^. 

However, a research conducted with 111 Nursing professionals from Alabama working on the front lines in the fight against COVID-19 pointed out that more than 54% reported that the supervisors had little concern for the professionals. However, the “supervisor support” variable was not associated with the “intention to leave the profession” outcome^20^.

In the current research, an association between presenting skin lesions and the intention to leave Nursing was observed. In contrast with the other factors that were associated with the intention to change professions, skin lesions are directly related to the COVID-19 pandemic context. As evidenced in the literature, this relationship is due to the increase in the PPE use time^26^.

A study carried out with Nursing professionals and physicians in a Chinese province found 97% prevalence of skin lesions due to PPE items used by front-line professionals in the fight against COVID-19^27^. Likewise, in Turkey, skin problems were found in 90.2%^28^ of the professionals. In turn, in a study conducted in the province of Wuhan (China), this prevalence was 91.8%^29^.

Skin lesions due to prolonged use of PPE items and to frequently hand washing or antisepsis were widely addressed in the COVID-19 pandemic context. However, up to the present day, no other studies have been identified in the literature associating skin lesions with the intention to leave the profession.

The association between skin lesions and the intention to leave the profession may have been due to subjective feelings in relation to the injuries, such as presence of pain, discomfort and infections. Lack of support in receiving products to adequately protect the skin, such as facial wipes, moisturizer and protective tape, is also pointed out. Protection of the professionals must be a priority in the health services to ensure that they can work without harms resulting from the use of equipment that, *a priori*, is intended to be protective^30^.

As a highly contagious and pathogenic disease, COVID-19 already took the lives of thousands of people, with many professionals among them. As evidenced, the fact that the professionals thought about leaving the profession was already a problem prior to the pandemic moment that exacerbated issues associated with overload and lack of institutional support at work^12^ ^,^ ^19^.

Although the pandemic has evidenced inadequate conditions and work overload, in addition to physical and mental risks, this research reinforces that deficient working conditions and lack of institutional support are factors that are not only present, in this pandemic moment. These factors were intensified in the pandemic context, overloading health services and leading Nursing professionals to physical and emotional exhaustion, contributing for them to think about changing professions.

The diverse information from this research contributes to advances in the subject matter, as it enables health service managers, as well as public sector, human resources and training schools managers to plan actions and strategies in order to reduce the number of Nursing professionals with an intention to leave the profession. In addition to this, it is important for the panorama of the profession, as it contributes to problematizing the need for improvements in the labor rights and working conditions, evidencing the importance of assessing the Nursing professionals’ needs.

It is to be pointed out that, for being a cross-sectional study, reverse causality cannot be disregarded. It is also noteworthy that the question used to track the outcome alluded to all the time elapsed since the beginning of the pandemic until the collection date, when the contagion curves were on an important rise. In this sense, it is important that future studies may strive to assess evolution of the outcome throughout the pandemic period, considering specific time clippings.

## Conclusion

High prevalence of the intention to leave Nursing was observed. Among the associated factors were sociodemographic aspects such as higher schooling levels (positive association) and age 51 years or older (negative association), in addition to structural issues such as overload and lack of institutional support (positive associations). In relation to the aspects directly related to the COVID-19 pandemic, it was possible to notice a number of associations between the outcome and the emergence of skin lesions (positive association). In this sense, it is pointed out that, except for skin lesions, there are issues among the modifiable factors that, although they may have been exacerbated by the pandemic, were already part of the daily routine of services, thus calling for services and health systems for the establishment of cross-sectional actions that can contribute to retention of Nursing professionals.
